# A case report: exudative retinal detachment secondary to combined infection of H1N1 influenza and *Mycoplasma*

**DOI:** 10.1186/s12348-026-00612-y

**Published:** 2026-06-10

**Authors:** Min Wu, Wenzhi Yang, Dan He, Zhuo Lang, Aoxue Yan, Geeta Menon, Gauri Narendran

**Affiliations:** 1 Ophthalmology Department, Affiliated Hospital of Yunnan University (The 2nd People’s Hospital of Yunnan Province, Yunnan Eye Hospital), Kunming, 650021 China; 2 Kunming Medical University, Kunming, 650000 China; 3 Frimley Health NHS Foundation Trust, Frimley, GU16 7UJ UK; 4 University of Oxford, Oxford, OX1 2JD UK

**Keywords:** Exudative retinal detachment, H1N1 influenza, Mycoplasma, High intraocular pressure, X-linked congenital retinoschisis

## Abstract

**Background:**

H1N1 influenza and Mycoplasma are common causes of pneumonia, but ocular symptoms after infection are rare. We report a case of bilateral decreased vision, exudative retinal detachment and high intraocular pressure secondary to H1N1 influenza and mycoplasma infection.

**Case presentation:**

A 7-year-old male presented with decreased vision in his right eye and was found to have exudative retinal detachment and high intraocular pressure secondary to combined infection of H1N1 influenza and mycoplasma. The boy was previously diagnosed with X-linked congenital retinoschisis (XLRS) at age 3. After anti-viral and anti-mycoplasma treatments, the exudative retinal detachment resolved, and the intraocular pressure was under control.

**Conclusions:**

This case highlights the possibility of exudative retinal detachment secondary to H1N1 influenza and mycoplasma, especially in patients with short axial length.

## Background

Exudative retinal detachment can be caused by inflammation, uveitis, tumours, systematic diseases and trauma. It has been reported that exudative retinal detachment could be an adverse event of H1N1 vaccination, with most such reports describing accompanying uveitis. It is also established that in cases of either H1N1 or Mycoplasma infection, similar complications have occurred on occasion. We present a 7-year-old patient with bilateral exudative retinal detachment and high intraocular pressure secondary to a combined infection of H1N1 influenza and mycoplasma.

## Case presentation

A 7-year-old boy presented with a one day history of painless decreased vision in his right eye. He had high hypermetropia of + 8 D and amblyopia, for which he had received amblyopia treatment since the age of 3 years. He also had bilateral retinoschisis associated with an X-linked gene mutation which was confirmed on genetic testing. The patient was born at 36 weeks by Caesarean section (G2P2). No neonatal asphyxia or resuscitation was reported. His mother had a history of gestational diabetes and hypertension.

**Fig. 1:**
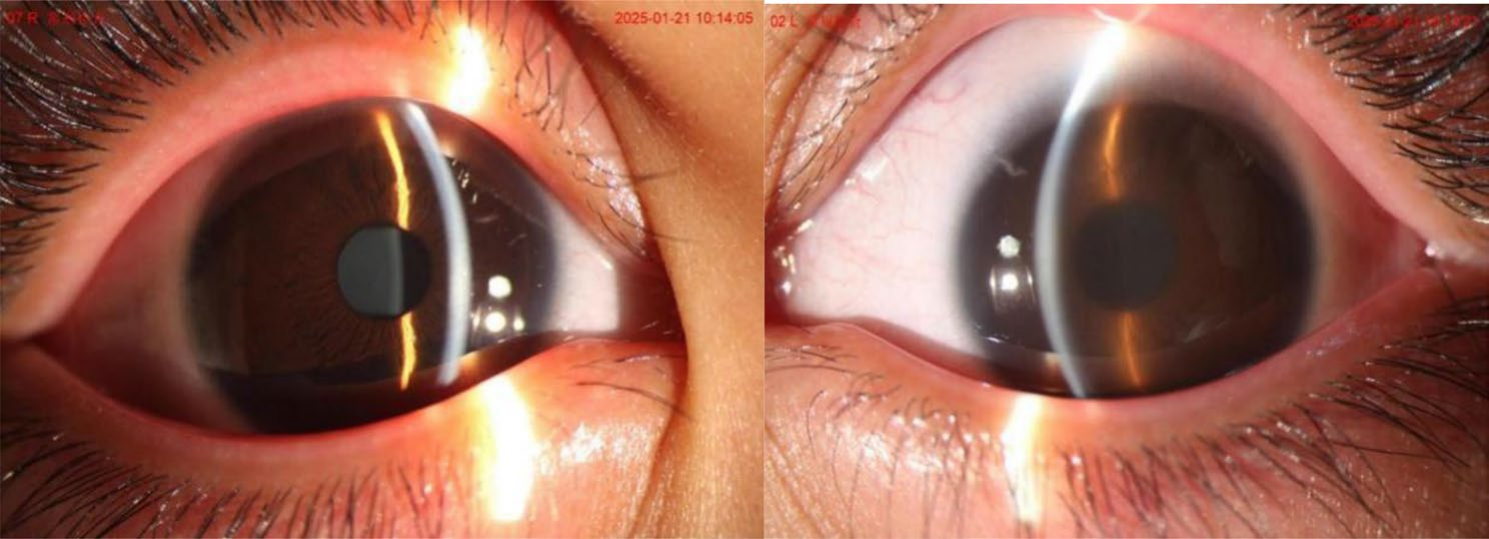
Anterior segment in both eyes

**Fig. 2:**
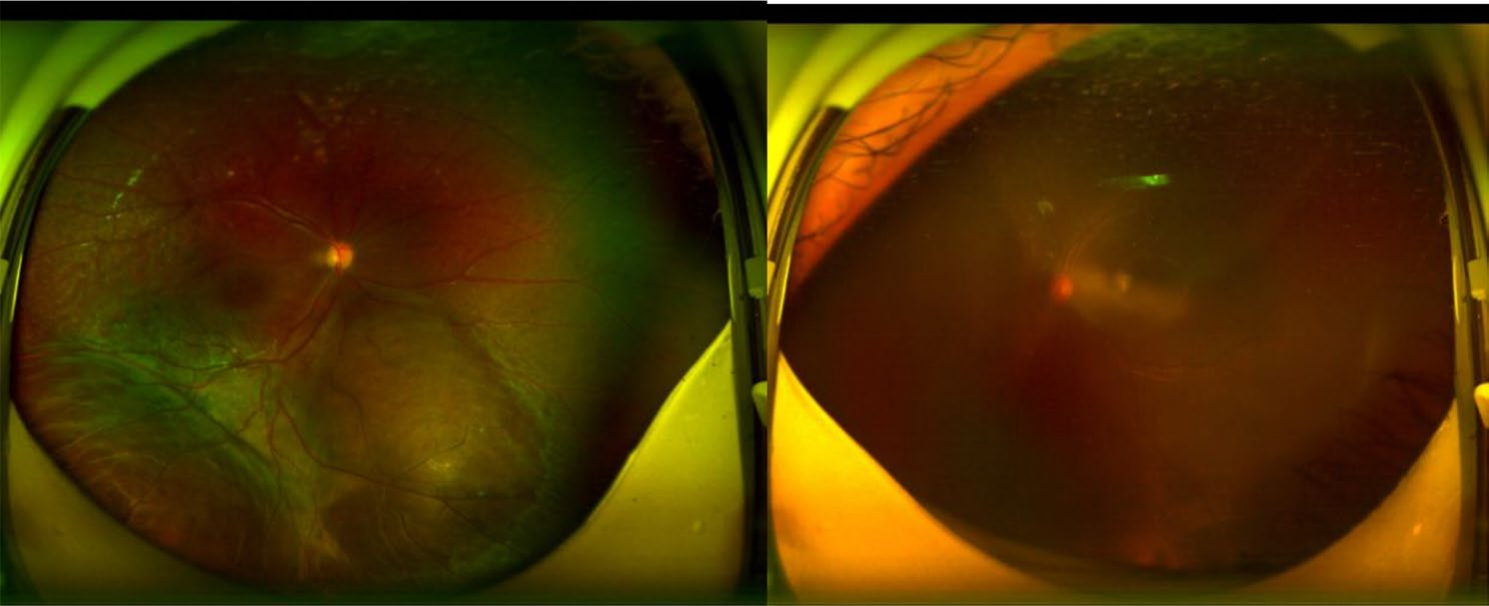
SLO

**Fig. 3:**
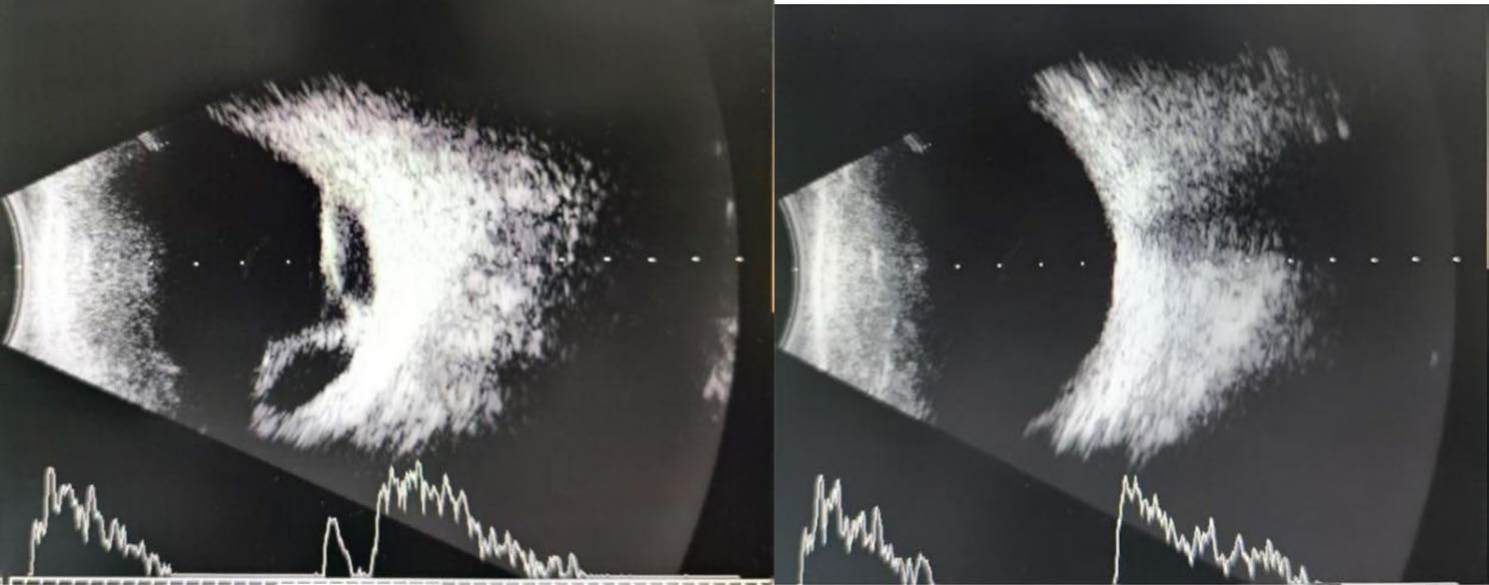
Ultrasound B scan

**Fig. 4:**
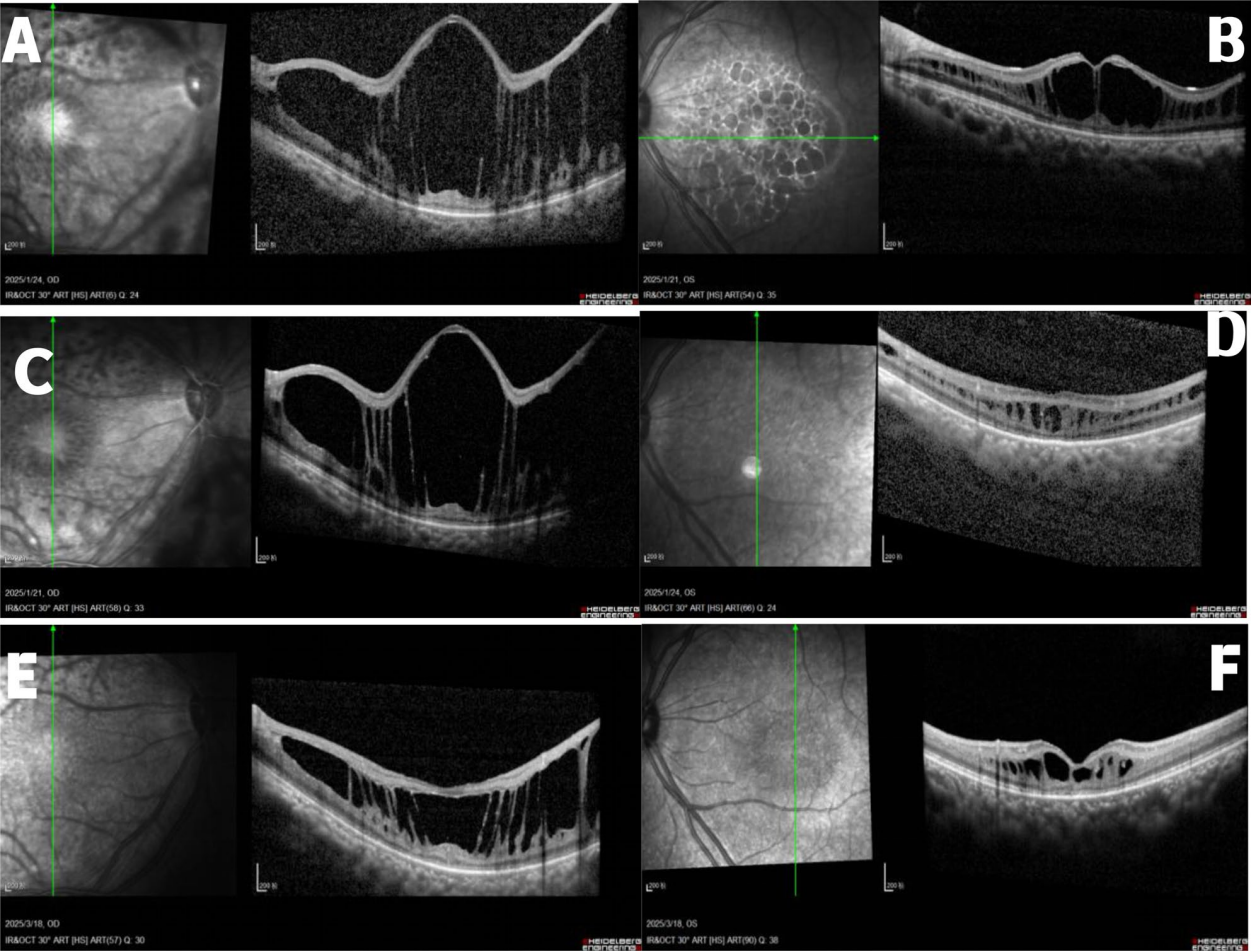
OCT scan for macula in three visits. **A** and **B**. Baseline OCT san; **C** and **D**. OCT scan after two days; **E** and **F**. OCT scan at last visit

**Fig. 5:**
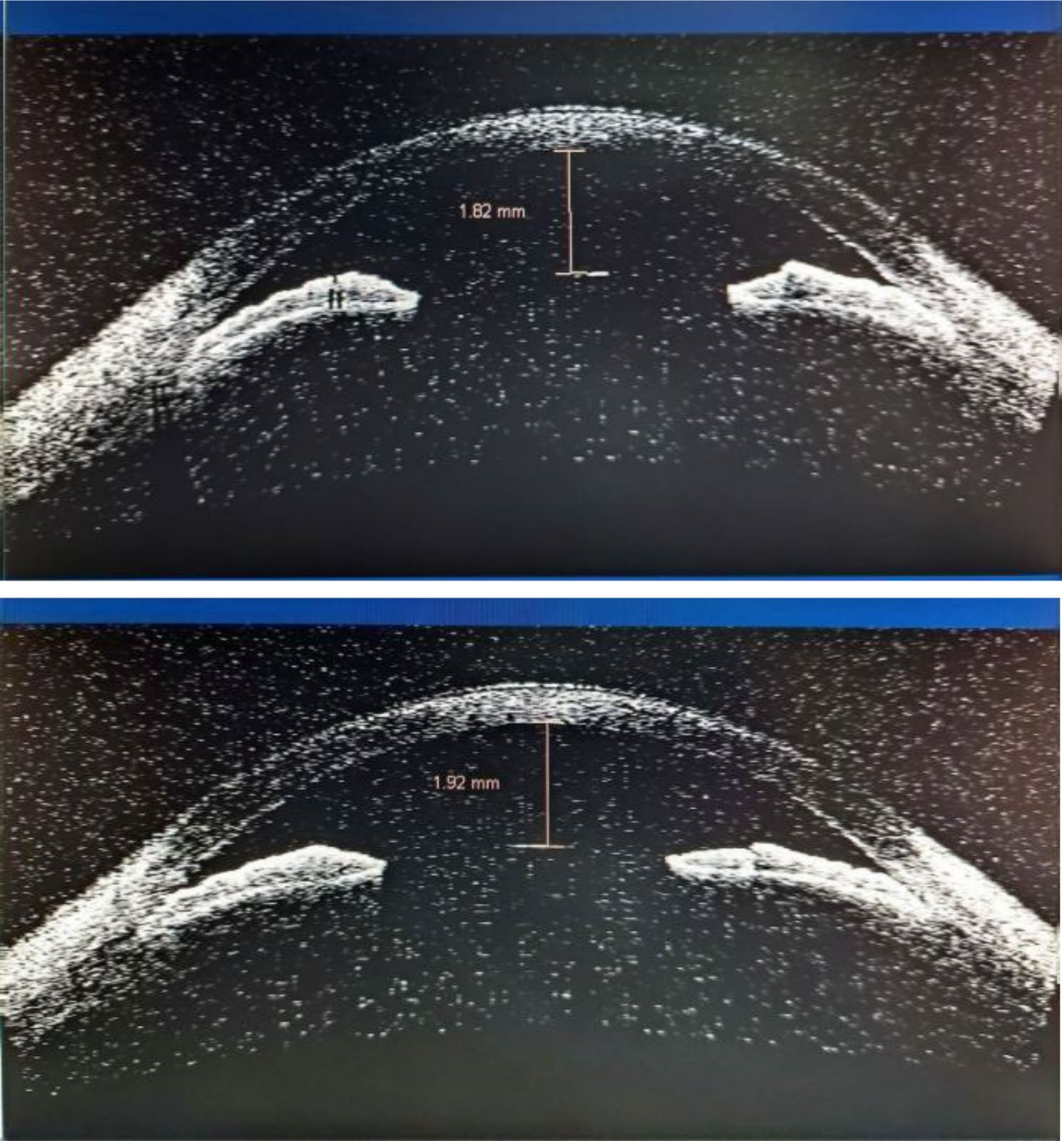
OCT scan for anterior segment. Anterior segment OCT showed shallow chamber in both eyes

Upon physical examination, there was visual acuity in the right eye (OD): finger counting, and in the left eye (OS): 20/125. Intraocular pressure (IOP) of OD 39 mmHg, OS 61 mmHg. The eyelids in both eyes were slightly swollen without tenderness. The cornea was clear in his right eye and corneal haze was noted in his left eye (see Fig. 1). Keratic precipitates (+) and anterior chamber cells (+) were noted in both eyes. Retinal detachment was noted without any retinal tears or holes in the inferior-temporal quadrant of his right eye. The retina of the left eye was flat (see Fig. 2). Ultrasound B scan confirmed that there was retinal detachment in the right eye (see Fig. 3). The axial length was 18.85 mm in the right eye and 19.55 mm in the left eye, measured by Ultrasound A scan. OCT scan showed bilateral macular retinoschisis (see Fig. 4). Anterior chamber depth (ACD) was 1.82 mm (OD) and 1.92 mm (OS)(see Fig. 5). The primary diagnosis included: Exudative retinal detachment in the right eye, with high intraocular pressure, X-linked retinoschisis and nanophthalmos in both eyes.

The mother said that the boy had the flu and a mycoplasma infection. In the local hospital, he was treated for two days with Azithromycin. The patient was sent to the paediatric department for further check-up, where he was diagnosed with pneumonia with H1N1, and mycoplasma test returning positive. H1N1 and mycoplasma test was conducted by PCR based on the serum specimens. The body temperature was 39.2 °C. Blood tests found that the white blood cell count was elevated to 13.17 × 10 g/L and platelet count was elevated to 544 × 10 g/L. C-reactive protein and procalcitonin were both in normal range.

CT scan showed mild bilateral scleral thickening, mild enlargement of the right lacrimal gland, and mild thickening of the right medial rectus, left lateral rectus, and bilateral inferior rectus muscles—suggestive of an inflammatory process. The patient was hospitalized in the paediatric department. Oseltamivir and Azithromycin were given orally and Prednisolone Acetate, atropine, Timolol and Brinzolamide eye drops were applied.

**Fig. 6:**
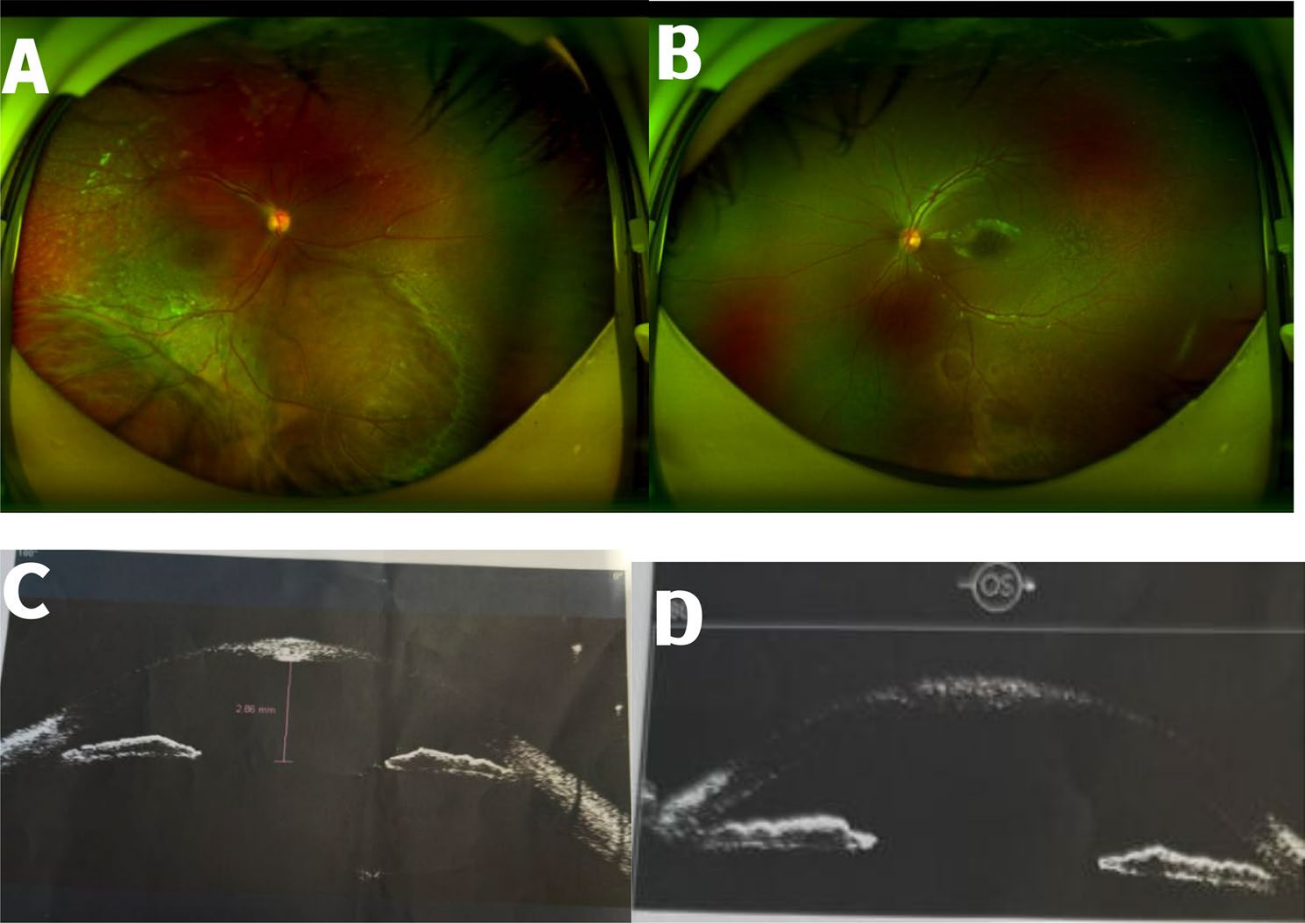
SLO and OCT scan after three days of treatment **A**. The SLO image in the right eye showed retinal detachment in the inferior part **B**. SLO image in left eye showed retinal detachment in the inferior-tumoral quadrant **C** and **D**. Anterior segment OCT scan in both eyes showed normal anterior chamber depth

The patient presented 2 days later in the ophthalmic clinic with decreased vision in his left eye. Best corrected visual acuity was finger counting in the right eye and 20/333 in the left eye. IOP was 28mmHg in the right eye (OD) and 35 mmHg in the left eye (OS). The left eye was noted to have developed a retinal detachment without any hole in the inferior-temporal quadrant (see Fig. 6). The retinal detachment in his right eye remained static. Dexamethasone 5 mg was administered intravenously for three days. One week later, the exudative retinal detachment was partially resolved.

Two months later, the best corrected visual acuity was 20/200 OD and 20/80 OS.

**Fig. 7:**
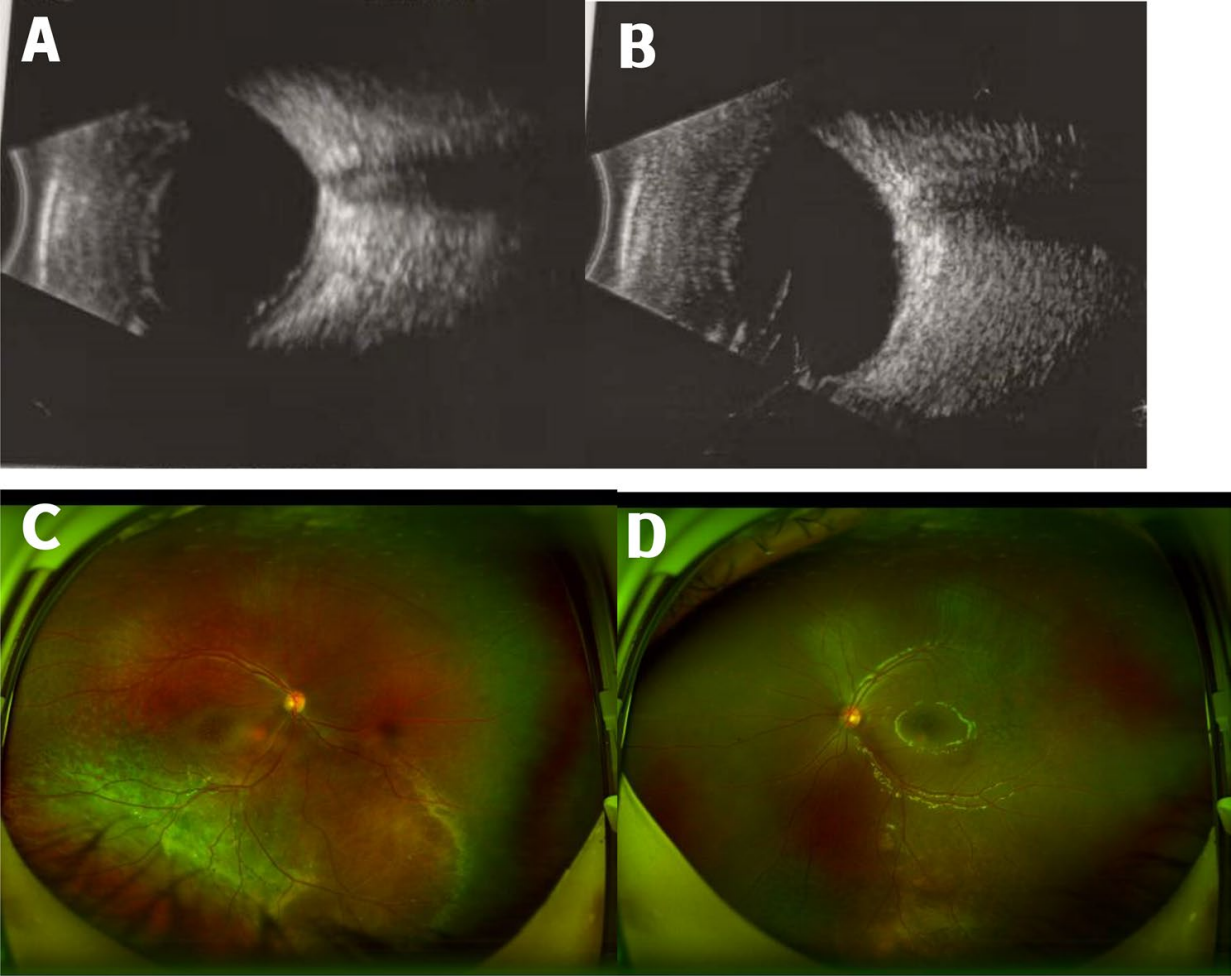
Examinations after two months. **A** and **B**. Ultrasound B-scan in both eyes showed retinal detachment **C**. SLO image in right eye showed area of retinal detachment in the inferior part reduced. **D**. The SLO image in the left eye showed a small area of retinal detachment in the inferior quadrant

IOP: OD 26mmHg and OS 31mmHg. The exudative retinal detachment had completely resolved in the left eye, but the right eye still had a small area of persistent exudative retinal detachment (see Fig. 7).Cup-disc ratio was 0.3 and RNFL was normal in both eyes.

## Discussion and conclusions

This case is a 7-year-old boy with a history of bilateral X-Linked retinoschisis, short axial length, hyperopia and amblyopia. He initially presented with exudative retinal detachment in right eye, mild anterior uveitis and high intraocular pressure in both eyes.

Systemic investigation indicated pneumonia caused by combined infection of H1N1 and mycoplasma. Three days later, exudative retinal detachment presented in his left eye. He was treated with anti-viral and anti-mycoplasma medication, steroids topically and intravenously, and IOP-lowering medication. After treatment, the exudative retinal detachment resolved completely in the left eye and partially in the right eye. After maximally tolerated medical therapy, IOP in both eyes at the last visit was lower than acute phase but still higher than normal. It seems that the long-term prognosis for him is generally poor and highly uncertain. The pre-existing structural fragility of the retina from XLRS, combined with persistent exudative detachment, refractory IOP elevation create dual challenges: difficulty in achieving anatomical reattachment and progressive visual function decline.

This was considered as an exudative retinal detachment secondary to H1N1 and mycoplasma infection. Differential diagnoses would include Uveal Effusion Syndrome (UES) and posterior scleritis. For this case, it is important to differentiate the exudative retinal detachment is caused by acute presentation from an exacerbation of UES triggered by systemic inflammation and a direct infectious/immune-mediated exudation from H1N1 and Mycoplasma. The child has significant baseline ocular pathologies, including nanophthalmos (axial length 18.85 mm OD, 19.55 mm OS) and XLRS. Patients with nanophthalmos could have exudative retinal detachment caused by Uveal Effusion Syndrome. The exudative retinal detachment related to UES usually occurs in adults with slow progression [1, 2]. Exudative retinal detachment caused by UES rarely occurs in children. For the acute presentation of UES with exudative retinal detachment, patients usually have normal or low IOP. Thickening sclera is a typical sign in UES. However, in our case, the IOP was high and mild bilateral scleral thickening was detected in CT scan. Furthermore, UES patients normally do not have inflammation in the anterior chamber and vitreous [1]. In our case, cells presented both in the anterior chamber and vitreous which supported the inflammation caused by H1N1 and Mycoplasma infection. Posterior scleritis has thickened posterior sclera and typical “T” sign evident on ultrasound B scan. In this case, no such signs were detected on ultrasound B scan.

Exudative retinal detachment can be caused by different diseases, such as VKH, tuberculosis, and various viral infections [3–6]. Some cases of exudative retinal detachment have been reported after H1N1 vaccination injection [3]. H1N1 infection has been reported in association with macular neuroretinopathy [7], Bilateral anterior uveitis, papillitis, neuroretinitis [8], Bilateral panuveitis [9] and Uveal effusion syndrome [10] in paediatric patients. H1N1 infection can cause autoimmune uveal inflammation and transient choroidal vascular leakage which contribute to choroidal effusion [11, 12]. Mycoplasma can result in immune complex-mediated posterior uveitis, choroidal oedema, elevated vascular permeability and cytokine-driven exudation [13]. Both H1N1 and Mycoplasma infection can trigger immune-mediated choroidopathy. It is possible that the combined infection led to more severe choroidal and retinal effusion in this patient. To the best of our knowledge, exudative retinal detachment caused by combined infection of H1N1 and mycoplasma has rarely been reported. Our patient has short axial length and congenital retinoschisis which made it more challenging. The exudative retinal detachment secondary to combined infection of H1N1 influenza and mycoplasma is rare and could be successfully treated with pharmacologic therapy. Anti-viral, anti-mycoplasma and steroid treatment induced resolution of the exudative retinal detachment in this patient.

The precise mechanism by which these immune mediated mechanisms manifest as ocular complications remains unclear. Many cases reporting such manifestations of H1N1 occur following vaccination, rather than infection (see Table 1) - comparing ocular findings in both scenarios may have important implications for the pathophysiology involved. It has been previously reported that ocular complications, particularly conjunctivitis, were far more common following H1N1 infection [12]. The same study noted uveal effusion syndrome in roughly 7.9% of those infected, and in none who were vaccinated - however, due to a small sample size, this wasn’t deemed a statistically significant difference between the two groups. Mycoplasma pneumoniae infection, on the other hand, is better known as a cause of systemic immune mediated disease, and ocular involvement following infection has been well documented [14].

Exudative retinal detachment occurs due to breakdown or disruption of the blood-retina barrier, which can occur because of an infectious or inflammatory process. H1N1 influenza may cause this through a variety of pathways. Most directly, infection of the vascular tissues themselves by the virus could cause the necessary damage to enable fluid leakage into the subretinal space. A recent in vitro study has shown that H1N1 is capable of infecting and replicating within human retinal microvascular endothelial cells (HRMECs), as well as upregulating proinflammatory factors including chemokines, cytokines, and interferon signalling amongst others [15]. This can disrupt the blood-retina barrier, and infected HRMECs showed rounding, detachment, and cell death - all of which facilitates fluid leakage and subretinal accumulation, leading to the ocular complications which are clinically observed.

However, this does not account for vaccine-related mechanisms where active infection is not necessarily present. Such cases, although rare, imply an immune response related pathophysiology - while no specific cause has been identified, there are several plausible mechanisms. This includes type III hypersensitivity (with immune complex deposition presumably within the retinal vasculature), adjuvant-induced inflammation, T-Cell activation, and autoimmune sensitization. There is, however, precedent for a single organism to be capable of causing ocular damage through both direct infection *and* post-infectious immune mechanisms - Mycobacterium tuberculosis can produce ocular manifestations both through infection of the ocular tissues and immune-mediated hypersensitivity reactions. Therefore, a similar set of mechanisms may be able to account for both the infection and vaccination-related H1N1 cases.

Contrastingly, in Mycoplasma related ocular complications, many cases present a number of days after symptom onset, or even after the initial infection has resolved [13]. This strongly indicates an immune-mediated response, which, in this context, is well established - M. pneumoniae is known to produce circulating immune complexes, which are capable of deposition in retinal vasculature, and to cause immune-mediated choroidal inflammation [14, 16]. Unlike H1N1 influenza, direct infection of ocular tissues is rarely observed in Mycoplasma-related cases, and the immune hypothesis is further strengthened by reports of steroid treatment resolving symptoms. While we cannot say for certain whether H1N1 or Mycoplasma were responsible for the ocular symptoms seen in our patient, their onset occurred within a similar timeframe to the infection itself, suggesting that a Mycoplasma-related pathology is less likely.

**Table 1:** Paediatric cases of exudative choroidal effusion related to H1N1 or *Mycoplasma* infection

Source / Year	Age / Sex	Trigger	Ocular Findings	Imaging / Investigations	Treatment & Outcome	Notes
Tao Y, et al. 2011, CMJ [3]	10 y boy & 47 y woman	H1N1 vaccination (10 days post-vaccine)	Acute uveitis with exudative retinal detachment (unilateral in child)	FA: multifocal pinpoint leaks; OCT: serous macular elevation	Topical + systemic corticosteroids; VA returned to 6/6 within 3 weeks	Highlights post-vaccination immune uveal reaction
Matsou A, et al. 2016, J AAPOS [12]	12 y girl	M. pneumoniae systemic infection	Bilateral panuveitis with serous detachment of posterior pole	OCT + FA: sub-retinal fluid; chest X-ray: interstitial pneumonia	Macrolide + oral prednisolone; complete resolution	Demonstrates immune-mediated posterior uveitis secondary to Mycoplasma
Liu and Janigian 2013, JAMA Ophthalmol [17]	14 y boy	Mycoplasma pneumoniae infection	Bilateral serous (exudative) macular detachment with optic disc swelling	OCT: sub-retinal fluid; FA: disc leakage; serology + PCR for M. pneumoniae	Azithromycin + oral corticosteroids → VA improved from 6/60 to 6/9	Classic adolescent case “The Other Masquerader”
Our case	7 year boy	H1N1 and Mycoplasma infection	Bilateral serous(exudative) retinal detachment and choroidal effusion, macular schisis, elevated IOP, short axial length, hyperopia, amblyopia	OCT, serous macular elevation, retinoschisis, narrow chamber B- scan: localized suprachoroidal fluid C- serology + PCR for Mycoplasma and H1N1	corticosteroids + Azithromycin+antiviral (oseltamivir); incomplete resolution in2 months	Combined infection of H1N1 and Mycoplasma

## Data Availability

No datasets were generated or analysed during the current study.

## Abbreviations

ACD: Anterior chamber depth
IOP: Intraocular pressure
OCT: Optical Coherence Tomography
XLRS: X-linked congenital retinoschisis
